# β-Cell-selective regulation of gene expression by nitric oxide

**DOI:** 10.1152/ajpregu.00240.2023

**Published:** 2024-04-08

**Authors:** Aaron Naatz, Chay Teng Yeo, Neil Hogg, John A. Corbett

**Affiliations:** ^1^Department of Biochemistry, Medical College of Wisconsin, Milwaukee, Wisconsin, United States; ^2^Department of Biophysics, Medical College of Wisconsin, Milwaukee, Wisconsin, United States

**Keywords:** β-cell, gene expression, islet, nitric oxide, reactive nitrogen species

## Abstract

Nitric oxide is produced at low micromolar levels following the induction of inducible nitric oxide synthase (iNOS) and is responsible for mediating the inhibitory actions of cytokines on glucose-stimulated insulin secretion by islets of Langerhans. It is through the inhibition of mitochondrial oxidative metabolism, specifically aconitase and complex 4 of the electron transport chain, that nitric oxide inhibits insulin secretion. Nitric oxide also attenuates protein synthesis, induces DNA damage, activates DNA repair pathways, and stimulates stress responses (unfolded protein and heat shock) in β-cells. In this report, the time- and concentration-dependent effects of nitric oxide on the expression of six genes known to participate in the response of β-cells to this free radical were examined. The genes included *Gadd45α* (DNA repair), *Puma* (apoptosis), *Hmox1* (antioxidant defense), *Hsp70* (heat shock), *Chop* (UPR), and *Ppargc1α* (mitochondrial biogenesis). We show that nitric oxide stimulates β-cell gene expression in a narrow concentration range of ∼0.5–1 µM or levels corresponding to iNOS-derived nitric oxide. At concentrations greater than 1 µM, nitric oxide fails to stimulate gene expression in β-cells, and this is associated with the inhibition of mitochondrial oxidative metabolism. This narrow concentration range of responses is β-cell selective, as the actions of nitric oxide in non-β-cells (α-cells, mouse embryonic fibroblasts, and macrophages) are concentration dependent. Our findings suggest that β-cells respond to a narrow concentration range of nitric oxide that is consistent with the levels produced following iNOS induction, and that these concentration-dependent actions are selective for insulin-containing cells.

## INTRODUCTION

Insulin-secreting β-cells are selectively destroyed during the development of insulin-dependent type 1 diabetes (T1D) through an autoimmune process (1, 2) where autoreactive cytotoxic T lymphocytes are responsible for most of the killing (2–5). Although genetic factors contribute to the development of T1D, the low concordance rates for disease development in identical twins support a role for environmental factors in the initiation of β-cell damage in this autoimmune disease (6, 7). Proinflammatory cytokines, such as interleukin (IL)-1, are also believed to contribute to the loss of β-cells during disease induction as treatment of rodent and human islets with cytokines results in an inhibition of insulin secretion and the loss of islet-cell viability following prolonged exposures (8, 9). IL-1 is the primary mediator of damage in rat islets whereas a combination of IL-1 and interferon (IFN)-γ are required to attenuate the function and decrease the viability of human islets (10, 11). The mechanisms by which cytokines damage β-cells have been extensively characterized. They inhibit insulin secretion in a concentration- and time-dependent manner with the initial inhibition observed following a 5- to 8-h incubation and complete inhibition following an 18-h incubation (8, 12). The inhibition of insulin secretion correlates with a simultaneous loss of mitochondrial oxidative capacity (aconitase and complex 4 of the electron transport chain) (13–16). Nitric oxide, produced at low micromolar levels (1–3 µM), following inducible nitric oxide synthase (iNOS) expression, is responsible for the inhibition of mitochondrial oxidative metabolism and insulin secretion (13–15, 17, 18). Nitric oxide also induces DNA damage (19–21), attenuates protein synthesis (22), and activates stress responses such as the unfolded protein response (UPR) and heat shock (22–25). DNA damage caused by nitric oxide results in the activation of the DNA damage response (DDR), which can result in DDR-dependent caspase-3 cleavage and apoptotic cell death following prolonged exposures (26).

Although these inhibitory and destructive actions of cytokines are believed to participate in the loss of functional β-cell mass during the development of autoimmune diabetes (27), we believe that there are physiological roles for cytokine signaling in islets that have yet to be fully elucidated. Importantly, islets represent ∼1–2% of the wet weight of the pancreas, yet receive 20% of pancreatic blood flow (28). Although this is expected for an endocrine hormone producing micro-organ that controls whole body glucose metabolism, it also necessitates that β-cells are exposed to all components of blood, including pyrogenic cytokines such as IL-1 and TNF, which are greatly elevated during infection (29). This would suggest that β-cells are routinely exposed to high levels of proinflammatory cytokines during infection, yet most individuals do not develop diabetes following infection. Furthermore, the inhibitory actions of cytokines on insulin secretion and mitochondrial oxidative metabolism are reversible (30, 31) and β-cells maintain pathways that facilitate the repair of DNA damage (32–35). Also, iNOS-derived levels of nitric oxide attenuate DDR activation, insulinoma cell apoptosis, and picornavirus replication (36–38). The inhibitory actions of nitric oxide on DDR activation and virus replication are selective for β-cells as they are not observed in non-β-cell populations (36, 37, 39–41). These findings suggest that there are physiological roles for cytokine-signaling and cytokine-induced nitric oxide production by β-cells.

The purpose of this report is to begin the process of understanding the concentration- and time-dependent actions of nitric oxide on the de novo expression of genes that play important physiological roles in the response of β-cells to cytokines. Because many of the protective actions of nitric oxide are cell type selective, we examined the effects of nitric oxide on gene expression in insulinoma cells, rat islets, and non-β-cell populations including α-cells (αTC1), mouse embryonic fibroblast (MEF) cells, and mouse macrophages (RAW 264.7). We focused our studies on genes regulated by the *1*) forkhead Box 1 (FoxO1) transcription factor, growth arrest, and DNA damage 45α (*Gadd45α*) and the proapoptotic factor p53 upregulated modulator of apoptosis (*Puma*) (35); *2*) nuclear regulatory factor 2 (NRF2), heme oxygenase-1 (*Hmox1*); *3*) heat shock response, heat shock protein 70 (*Hsp70)*; *4*) unfolded protein response, C/EBP homologous protein (*Chop*); and *5*) transcriptional coactivator, peroxisome proliferator-activated receptor γ coactivator 1-α (*Ppargc1α*), which functions in mitochondrial biogenesis (42, 43). Overall, we show that β-cell expression of these genes is limited to a narrow steady-state concentration of nitric oxide between 0.5 and 1 µM. When supplied at concentrations greater than 1 μM, gene expression is delayed until the levels decrease back into this narrow range. Furthermore, these narrow concentration-dependent actions of nitric oxide are limited to insulin-producing cells. There are no delays in nitric oxide-dependent gene expression in non-β-cells at concentrations greater than 1 µM, and nitric oxide fails to stimulate the expression of several of the target genes in noninsulin-containing cells. These exciting findings provide experimental evidence supporting that there is a physiological range of nitric oxide production (∼0.5–1 µM, corresponding to iNOS-derived levels) that regulates gene expression in a β-cell-selective manner.

## MATERIALS AND METHODS

### Materials

(*Z*)-1-(*N*,*N*-Diethylamino)diazen-1-ium-1,2-diolate (DEA/NO) (No. 82100), (*Z*)-1-[*N*-(3-aminopropyl)-*N*-(3-ammoniopropyl)amino]diazen-1-ium-1,2-diolate (DPTA/NO) (No. 82110), and 2-(4-carboxyphenyl)-4,5-dihydro-4,4,5,5-tetramethyl-1*H*-imidazolyl-1-oxy-3-oxide (CPTIO) potassium salt (No. 81540) were purchased from Cayman Chemical (Ann Arbor, MI). RPMI 1640 medium, Dulbecco’s modified eagle medium (DMEM), trypsin, l-glutamine, penicillin, and streptomycin were purchased from Corning (Corning, NY). CMRL medium, sodium pyruvate, HEPES, and β-mercaptoethanol were from Thermo Fisher Scientific (Waltham, MA). Fetal bovine serum was obtained from Hyclone Laboratories (Logan, UT). INS 832/13 cells were a gift from Chris Newgard (Duke University, Durham, NC). RAW 264.7 cells were procured from the Washington University Tissue Culture Support Center (St. Louis, MO). MEF (CRL-2991) and αTC1 clone 6 (CRL-2934) cells were purchased through the American Type Culture Collection (Manassas, VA). Male Sprague-Dawley rats were purchased from Envigo (Indianapolis, IN).

### Cell Culture

INS 832/13, MEF, αTC1, and RAW 264.7 cell lines were cultured at 37°C with 5% atmospheric CO_2_ as described previously (36, 38). Cells were detached from culture flasks with 0.05% trypsin in 0.53 mmol/L EDTA and plated at 500,000 cells/mL (INS 832/13, αTC1, and RAW 264.7) or 125,000 cells/mL (MEF) and cultured overnight. All cell types used in this study are routinely tested and are mycoplasma free.

### Rat Islet Isolation

Islets were isolated from male Sprague-Dawley rats, cultured at 37°C with 5% atmospheric CO_2_ (44). Islets were dispersed into individual cells by trypsin digestion (45) and cultured for an additional 2 days before treatment. Animal care and procedures with rats were approved by the Institutional Animal Care and Use Committees at the Medical College of Wisconsin (A3102-01).

### qPCR

RNA from whole cell lysates was isolated using the RNeasy mini kit (Qiagen, Valencia, CA) and treated with Turbo DNase (Thermo Fisher). After RNA incubation with oligo(dT)_20_ (Integrated DNA Technologies, Coralville, IA), first-strand cDNA synthesis was performed using Maxima H minus reverse transcriptase (Thermo Fisher). cDNA was treated with RNase H (New England Biolabs, Ipswich, MA). Quantitative real-time PCR using SsoFast EvaGreen supermix was run using the CFX96 Real Time System (Bio-Rad, Hercules, CA). Fold changes in gene expression were measured by the ΔΔCT method (46), with values normalized to *Gapdh*. Primers used for qPCR are shown in Table 1 and were purchased from Integrated DNA Technologies.

**Table 1.** qPCR primers

Gene	Species	Primer	Sequence 5′-3′
*Chop*	Mouse/Rat	For Rev	AAATAACAGCCGGAACCTGA GGGATGCAGGGTCAAGAGTA
*Gadd45*α	Mouse	For Rev	GCAGAGTTCCCCAGCGAGGC GCCCACCGTGTCCATCCTTTCG
*Gadd45*α	Rat	For Rev	GTGTGCTGGTGACGAACCCACAT CCGTTCGGGGAATCACCGTCCG
*Gapdh*	Mouse/Rat	For Rev	GACATCAAGAAGGTGGTGAAGC TCCAGGGTTTCTTACTCCTTGG
*Hmox1*	Mouse	For Rev	CTCGAGCATAGCCCGGAGCC ATCCTGGGGCATGCTGTCGGG
*Hmox1*	Rat	For Rev	CGCCTCCAACCAGCGAGTGG GGACATGCTGTCGAGCTGTGGG
*Hsp70*	Mouse/Rat	For Rev	CATGAAGCACTGGCCCTTCC CGAAGATGAGCACGTTGCGC
*Ppargc1*α	Mouse/Rat	For Rev	AGTCCCATACACAACCGCAGTCG GGGGAACCCTTGGGGTCATTTGG
*Puma*	Mouse/Rat	For Rev	GAAGAGCAACATCGACACCG GGTGTAGGCACCTAGTTGGG

### Estimation of Steady-State Nitric Oxide Concentration

Steady-state nitric oxide concentrations released from the nitric oxide donor compounds DEA/NO and DPTA/NO were estimated using Gepasi 3.30 Biochemical Simulation software using calculations described by Mokry et al. (47). The first-order decay rates for DEA/NO and DPTA/NO at 37°C were 3.851 × 10^−3^·s^−1^ (*t*_1/2_ ∼3 min) and 6.418 × 10^−5^·s^−1^ (*t*_1/2_ ∼180 min), and moles of nitric oxide released per mole of donor compound were 1.5 (DEA/NO) and 2 (DPTA/NO), respectively (48). The oxygen concentration was fixed at 220 μM, with the rate of nitric oxide and oxygen reacting to form NO_2_ set to 2.4 × 10^6^ M^−2^·s^−1^ (49). The rate used for reaction of nitric oxide with NO_2_ to form nitrite was 1.1 × 10^9^ M^−2^·s^−1^ (50).

### Measurement of Steady-State Levels of Nitric Oxide

A Sievers 280i NO analyzer (GE Analytical Instruments) was used to determine the steady-state nitric oxide concentrations released from DPTA/NO. DPTA/NO was added to prewarmed INS media that was kept at 37°C for the duration of the experiment. Immediately following donor addition, INS media was put under a stream of 5% CO_2_-95% air for 15 s and capped to maintain pH 7.4. Chemiluminescent signal (mV) was measured for each sample, followed by peak integration and area determination. Final nitric oxide concentrations were calculated based on a nitrite standard curve (51).

### Cellular Bioenergetics

A Seahorse XFe96 analyzer (Agilent Technologies) was used to measure oxygen consumption rate (OCR) and extracellular acidification rate (ECAR) in MEF (10,000 cells/well) and INS 832/13 (20,000 cells/well) as outlined previously (40). Extracellular flux measurements were made in DMEM that contained 5.5 mM glucose, 2 mM pyruvate, and 1 mM l-glutamine. Values were expressed as percent baseline according to each cell type.

### Statistical Analysis

One-way ANOVA was performed for experimental results and statistical significance (*P* < 0.05) was determined using a Tukey’s multiple comparisons post hoc test.

## RESULTS

### Concentration-Dependent Effects of Exogenous Nitric Oxide Released from DEA/NO on β-Cell Gene Expression

**Figure 1.**
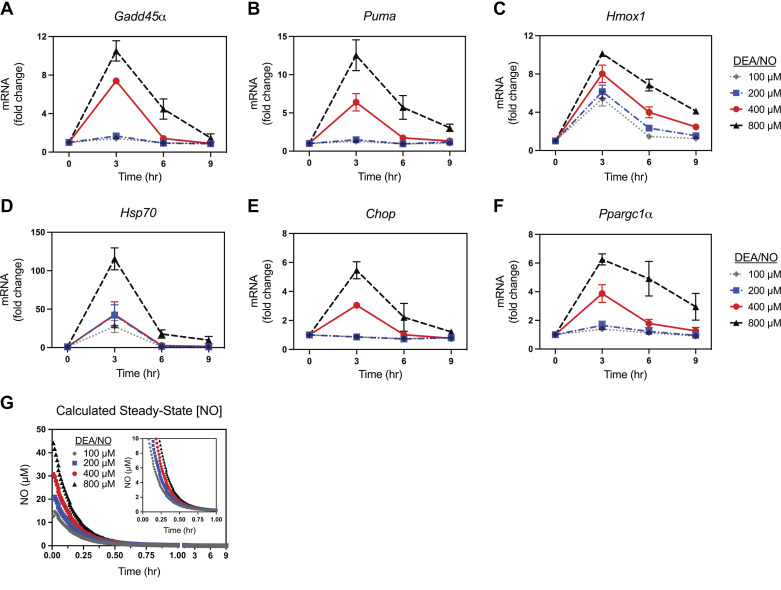
Concentration- and time-dependent effect of (*Z*)-1-(*N*,*N*-diethylamino)diazen-1-ium-1,2-diolate (DEA/NO) on gene expression in INS 832/13 cells. INS 832/13 cells were treated with DEA/NO for the indicated times and concentrations. The cells were harvested, and mRNA accumulation was determined by RT qPCR for *Gadd45α* (*A*), *Puma* (*B*), *Hmox1* (*C*), *Hsp70* (*D*), *Chop* (*E*), and *Ppargc1α* (*F*). Calculated steady-state nitric oxide concentrations released from DEA/NO over 9 h (*G*) and during the first h (*G*, *inset*) are shown. Results are the means ± SE of three independent experiments (*A–F*).

Although nitric oxide, derived from iNOS, is known to mediate the inhibitory actions of cytokines (IL-1 and IFN-γ) on insulin secretion by rodent and human β-cells (11, 14, 20), much less is known about the regulation of genes whose expression is induced by this free radical. We have identified several genes whose expression is stimulated by nitric oxide and have recently confirmed their expression by single-cell RNAseq analysis of cytokine-treated mouse islets (52). Each of the genes examined are known to participate in the physiological responses of β-cells to cytokine-induced nitric oxide (24, 33, 35, 38, 53, 54). Initial experiments examined the time- (0–9 h) and concentration- (100–800 µM) dependent actions of nitric oxide generated from the nitric oxide donor DEA/NO on changes in gene expression of INS 832/13 cells. As shown in Fig. 1, DEA/NO stimulates the concentration-dependent mRNA accumulation of the DNA base excision repair (BER) gene *Gadd45α*, the proapoptotic gene *Puma*, the antioxidant Nrf2-dependent gene *Hmox1* (Fig. 1, *A*–*C*), the heat shock response gene *Hsp70*, the unfolded protein response regulator *Chop*, and the mitochondrial biogenesis gene *Ppargc1α* (Fig. 1, *D*–*F*). For each of these genes, maximal mRNA accumulation was observed following a 3-h exposure to DEA/NO with the steady-state levels of each mRNA returning to near control levels by 9 h. *Hmox1* and *Hsp70* responded to all concentrations of DEA/NO examined, whereas *Gadd45α*, *Puma*, *Chop*, and *Ppargc1α* mRNA accumulation was observed in response to concentrations of 400 µM and 800 µM donor. DEA/NO has a short half-life (∼3 min) (48) and rapidly releases high levels of nitric oxide within minutes of treatment. The calculated steady-state nitric oxide concentrations are more than 10 µM early in the treatment (15 min) and fall to 1–2 µM following a 30-min incubation at each concentration (Fig. 1*G*). The calculated steady-state levels of nitric oxide fall below micromolar concentrations by 45 min (Fig. 1*G*, *inset*).

**Figure 2.**
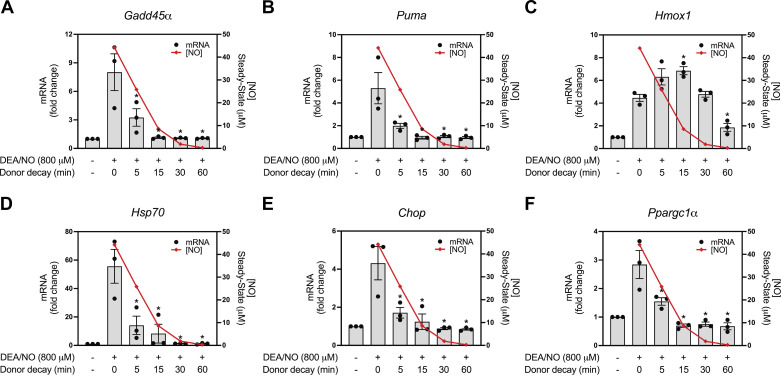
Time-dependent effect of decayed (*Z*)-1-(*N*,*N*-diethylamino)diazen-1-ium-1,2-diolate (DEA/NO) on nitric oxide-stimulated gene expression in INS 832/13 cells. DEA/NO (800 µM) was added to prewarmed INS media for 0, 5, 15, 30, or 60 min to release a portion of nitric oxide. After this decay period, the media was added to INS832/13 cell followed by a 3-h incubation. The cells were harvested and nitric oxide-stimulated mRNA accumulation of *Gadd45α* (*A*), *Puma* (*B*), *Hmox1* (*C*), *Hsp70* (*D*), *Chop* (*E*), and *Ppargc1α* (*F*) were evaluated by RT qPCR (*left Y-*axis). The steady-state concentrations of nitric oxide released from DEA/NO over 60 min were calculated (*A–F*, *right Y*-axis). Results are the means ± SE of three independent experiments (*A–F*). Statistically significant changes in mRNA accumulation compared with 0 min DEA/NO decay are indicated (*P < 0.05).

To determine if there is a threshold level of nitric oxide that is required to stimulate gene expression, DEA/NO was preincubated in media for various times between 0 and 60 min to release nitric oxide before treatment of INS 832/13 cells. Using this approach, a 15-min preincubation of DEA/NO is equivalent to five half-lives of donor decay. As shown in Fig. 2, 800 μM DEA/NO was directly added to cells (0 min) or was preincubated (5, 15, 30, 60 min) in media and then added to INS 832/13 cells followed by three additional hours of culture. The cells were harvested, and steady-state mRNA levels of each target were determined by qPCR (Fig. 2, bar graph) and compared with the calculated concentrations of nitric oxide (Fig. 2, line graph). The direct addition of DEA/NO to INS 832/13 cells (0 min) stimulates an increase in the expression for all genes as seen previously in Fig. 1 (Fig. 2, *A*–*F*). After a 5-min decay period, or ∼2 half-lives of donor decay, there was a significant reduction in the accumulation of *Gadd45α*, *Puma*, *Hsp70*, *Chop*, and *Ppargc1α* mRNA when compared with the direct addition of DEA/NO (0 min). This occurs under conditions where the calculated steady-state nitric oxide concentration is expected to be ∼25 μM (Fig. 2, *A* and *B*, and *D*–*F*). After a 15-min decay of DEA/NO, there is a significant attenuation in the accumulation of each of the mRNAs except *Hmox1*, which accumulates to near-maximal levels. Also, and to a lesser extent, *Hsp70* is still induced following a 15-min decay of DEA/NO (∼10-fold; Fig. 2*D*). After a 60-min pretreatment where the calculated steady-state concentration of nitric oxide falls below micromolar levels (∼0.3 μM, Fig. 1*G*, *inset*), DEA/NO does not stimulate mRNA accumulation of any of the genes examined. Overall, the findings presented in Figs. 1 and 2 indicate that heat shock/antioxidant gene expression is more responsive to nitric oxide than genes involved in DNA repair and stress responses, and that low micromolar levels of nitric oxide are required to stimulate new gene expression.

### Concentration-Dependent Effects of Exogenous Nitric Oxide Released from DPTA/NO on β-Cell Gene Expression

**Figure 3.**
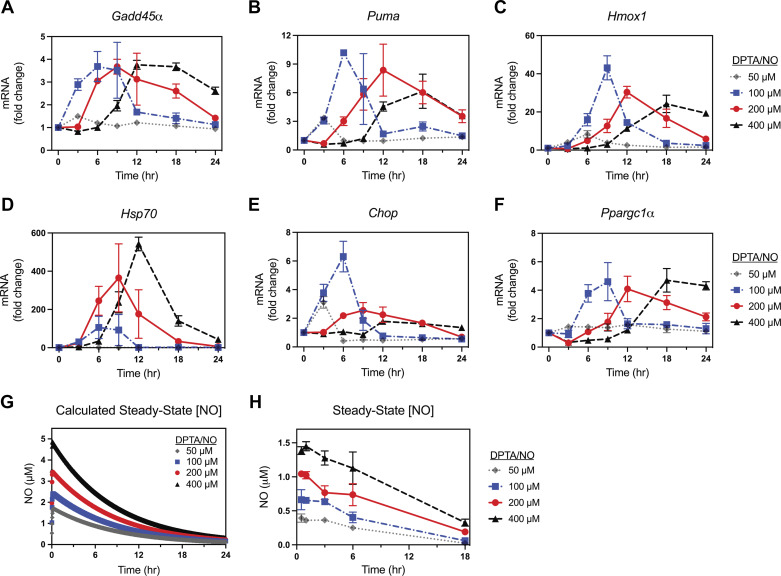
Concentration- and time-dependent effect of (*Z*)-1-[*N*-(3-aminopropyl)-*N*-(3-ammoniopropyl)amino]diazen-1-ium-1,2-diolate (DPTA/NO) on gene expression in INS 832/13 cells. INS 832/13 cells were treated for 3 to 24 h with the indicated concentrations of DPTA/NO. The cells were harvested, and mRNA accumulation was determined by RT qPCR for *Gadd45α* (*A*), *Puma* (*B*), *Hmox1* (*C*), *Hsp70* (*D*), *Chop* (*E*), and *Ppargc1α* (*F*). The calculated steady-state levels of nitric oxide released from DPTA/NO over 24 h are shown (*G*). The steady-state levels of nitric oxide released from DPTA/NO were measured using the NO analyzer following 0.5–18 h incubation in INS media (*H*). Results are the means ± SE of three independent experiments (*A–F* and *H*).

Results presented in Figs. 1 and 2 suggest that low micromolar levels of nitric oxide are required for the induction of gene expression in β-cells; however, this approach was limited by the rapid rates of nitric oxide released from this short half-life donor. To address this limitation, a second nitric oxide donor, DPTA/NO, with a 3-h half-life was used (48). DPTA/NO provides for sustained release of nitric oxide in the low micromolar range or physiological concentrations that are produced in β-cells expressing iNOS (13, 14). INS 832/13 cells were treated with increasing concentrations of DPTA/NO from 50 to 400 μM for 3–24 h and mRNA accumulation for each of the six genes was determined by qPCR. At 50 µM, new gene expression in response to DPTA/NO is minimal, reaching a twofold or greater increase in *Puma*, *Hmox1*, *Hsp70*, and *Chop* mRNA following a 3-h incubation (Fig. 3, *B–E*). Increasing the concentration of DPTA/NO to 100 μM results in an increase in the mRNA accumulation of all genes examined in a time-dependent manner that is first apparent at 3 h and maximal at 6–9 h. The maximal fold increases in mRNA accumulation are as follows: *Gadd45α,* 3.5-fold; *Puma,* 10-fold; *Hmox1*, ∼40-fold; *Hsp70*, ∼100-fold; *Chop*, 6.5-fold; and *Ppargc1α*, 4.5-fold (Fig. 3, *A–F*). Increasing the concentration of DPTA/NO to 200 µM and 400 μM results in a delay in the induction of mRNA accumulation of all the genes as well as an increase in the incubation time required to stimulate maximal mRNA accumulation (Fig. 3, *A–F*); however, the maximal level of mRNA accumulation is similar for most of the genes examined except for the stress response genes *Hsp70* and *Chop*. *Hsp70* mRNA accumulation is concentration-dependent and maximal at 400 μM DPTA/NO, whereas *Chop* mRNA accumulation is maximal at 100 µM DPTA/NO and decreases with increasing concentrations of the donor (Fig. 3, *D* and *E*). Overall, these data suggest that there is a window of sustained steady-state production of nitric oxide in the high nanomolar to low micromolar range that is capable of stimulating gene expression in INS 832/13 cells.

Two methods were used to gain insights into the amount of nitric oxide liberated by DPTA/NO. We estimated concentration of nitric oxide released by DPTA/NO using Gepasi 3.30 Biochemical Simulation (Fig. 3*G*) and measured the levels released by ozone-chemiluminescence using a Sievers nitric oxide analyzer (Fig. 3*H*). Overall, the calculated levels of nitric oxide were approximately three times higher than the directly measured levels, but the overall levels fall in the range generally associated with levels of nitric oxide produced following iNOS induction. Specifically, the steady-state concentration of nitric oxide produced in response to 50 μM DPTA/NO at 3 h was calculated to be ∼1–1.5 µM or measured to be ∼0.5 µM, and this concentration is sufficient to initiate mRNA accumulation for most of the genes examined. Increasing DPTA/NO to 100 µM results in the liberation of steady-state nitric oxide levels at 1.5–2.5 µM calculated or 0.5–0.7 µM measured, during the first 3 h of incubation. Increasing DPTA/NO levels to 200 µM and 400 µM results in steady-state levels of nitric oxide generation greater than ∼2.5 µM calculated or ∼0.8 µM measured during the first 3 h of incubation, or concentrations that appear to exceed the levels sufficient to support nitric oxide-dependent gene expression. As the incubation time is increased to 6 h and 9 h, the steady-state levels of nitric oxide that are released fall to levels (1.5–2.5 µM calculated or 0.5–1 µM measured) that can support gene expression.

**Figure 4.**
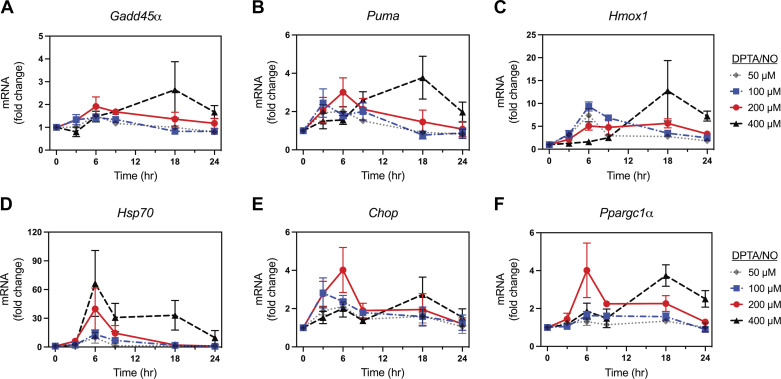
Concentration- and time-dependent effect of (*Z*)-1-[*N*-(3-aminopropyl)-*N*-(3-ammoniopropyl)amino]diazen-1-ium-1,2-diolate (DPTA/NO) on gene expression in dispersed rat islet cells. Isolated rat islets were dispersed into individual cells and then treated for 3–24 h with the indicated concentrations of DPTA/NO. The cells were harvested, and mRNA accumulation was determined by RT qPCR for *Gadd45α* (*A*), *Puma* (*B*), *Hmox1* (*C*), *Hsp70* (*D*), *Chop* (*E*), and *Ppargc1α* (*F*). Results are the means ± SE of three to five independent experiments.

### Concentration- and Time-Dependent Induction of Gene Expression by DPTA/NO in Rat Islet Cells

Using a similar approach, the time- and concentration-dependent effects of DPTA/NO on gene expression were explored in primary dispersed rat islet cells (Fig. 4). Much like INS 832/13 cells, there is a less than twofold increase in the accumulation of mRNA for all genes examined in response to 50 μM DPTA/NO, except *Hmox1* and *Hsp70* (Fig. 4, *A*–*F*). Increasing the concentration of DPTA/NO results in an increase in the mRNA accumulation of each gene examined (Fig. 4, *A*–*F*). Although *Puma* and *Chop* mRNA accumulate following a 3-h incubation (Fig. 4, *B* and *E*), induction of *Gadd45α*, *Hmox1*, *Hsp70*, and *Ppargc1α* require a longer 6-h incubation with DPTA/NO at 100 µM and 200 µM to stimulate maximal mRNA accumulation (Fig. 4, *A*, *C*, *D*, and *F*). When DPTA/NO is provided at 400 μM, the accumulation of each of the mRNAs is delayed with maximal mRNA accumulation of *Gadd45α* (2.5-fold), *Puma* (∼4-fold), *Hmox1* (∼13-fold), *Chop* (∼2.5-fold), and *Ppargc1α* (3.5-fold) occurring following an 18-h incubation (Fig. 4, *A*–*C*, *E*, and *F*). *Hsp70* mRNA accumulation was concentration dependent with peak accumulation at 6 h and remained elevated following an 18-h incubation with 400 µM DPTA/NO (Fig. 4*D*). Like the response of INS 832/13 cells, these findings indicate that iNOS-derived physiological concentrations of nitric oxide (1.5–2.5 µM calculated or 0.5–1 µM measured) facilitate gene expression in primary islet cells.

### Buffering Nitric Oxide Levels Using CPTIO

**Figure 5.**
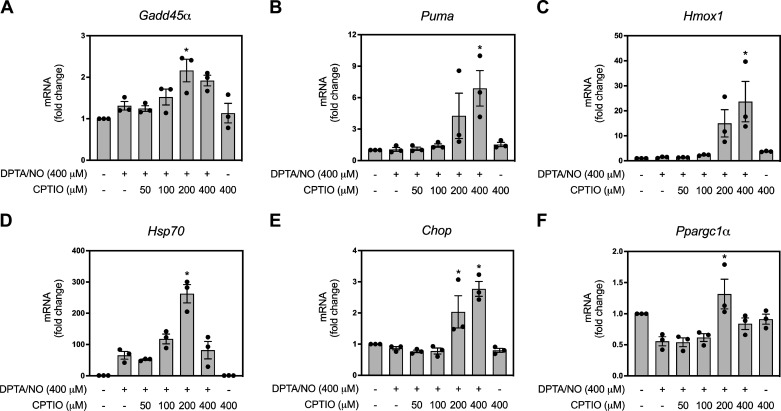
Effects of the nitric oxide scavenger 2-(4-carboxyphenyl)-4,5-dihydro-4,4,5,5-tetramethyl-1*H*-imidazolyl-1-oxy-3-oxide (CPTIO) on (*Z*)-1-[*N*-(3-aminopropyl)-*N*-(3-ammoniopropyl)amino]diazen-1-ium-1,2-diolate (DPTA/NO) stimulated gene expression in INS 832/13 cells. INS 832/13 cells were treated for 6 h with 400 μM DPTA/NO in the presence or absence of the indicated concentrations of CPTIO. The cells were harvested, and mRNA accumulation was determined by RT qPCR for *Gadd45α* (*A*), *Puma* (*B*), *Hmox1* (*C*), *Hsp70* (*D*), *Chop* (*E*), and *Ppargc1α* (*F*). Results are the means ± SE of three independent experiments and statistically significant changes in mRNA accumulation compared with the levels determined following a 400-µM treatment with DPTA/NO are indicated (*P < 0.05).

The results presented in Figs. 1–4 suggest that there is a narrow concentration window in which nitric oxide stimulates gene expression in β-cells. To confirm these surprising results, we devised a second approach to examine whether this narrow but physiologically relevant iNOS-derived level of nitric oxide controls gene expression in β-cells. We reasoned that it should be possible to buffer the level of nitric oxide released from high concentrations of DPTA/NO using increasing concentrations of the nitric oxide scavenger, CPTIO. In this experiment, INS 832/13 cells were treated for 6 h with 400 μM DPTA/NO in the presence or absence of increasing concentrations (50–400 μM) of CPTIO (Fig. 5). DPTA/NO at 400 µM fails to stimulate the accumulation of *Gadd45α*, *Puma*, *Hmox1*, *Chop*, or *Ppargc1α* mRNA in INS 832/13 cells following a 6-h incubation (Fig. 5, *A*–*C*, *E*, and *F*), consistent with the effects of 400 µM DPTA/NO shown in Fig. 3, *A*–*F*. Under these conditions, the steady-state levels of nitric oxide liberated from this donor exceed the levels necessary for the induction of gene expression in INS 832/13 cells (Figs. 3*G* and 4*H*). If this interpretation is correct, then it should be possible to scavenge nitric oxide produced by DPTA/NO to levels that allow for gene expression. CPTIO functions as a stoichiometric scavenger of nitric oxide (55), whereas DPTA/NO releases two molecules of nitric oxide per molecule of donor (48). In the presence of 200 µM and 400 µM CPTIO, DPTA/NO stimulates a twofold increase in *Gadd45α*, 4- to 7-fold increase in *Puma*, 15- to ∼25-fold increase in *Hmox1*, and 2- to 3-fold increase in *Chop* mRNA accumulation (Fig. 5, *A*–*C*, and *E*). Furthermore, DPTA/NO (400 µM) alone stimulates *Hsp70* mRNA accumulation following a 6-h treatment, and this is increased to ∼250-fold when cotreated with 200 μM CPTIO (Fig. 5*D*). *Ppargc1α* mRNA accumulation is decreased to 0.5-fold of basal with 400 μM DPTA/NO treatment, and cotreatment with 200 μM CPTIO returned *Ppargc1α* mRNA levels to slightly higher than those observed under basal conditions (Fig. 5*F*). Overall, these results support a narrow concentration range of between 1.5 and 2.5 µM calculated or 0.5–1 µM measured in which nitric oxide stimulates gene expression in β-cells.

### Cell Type Specificity of Nitric Oxide-Dependent Gene Expression

**Figure 6.**
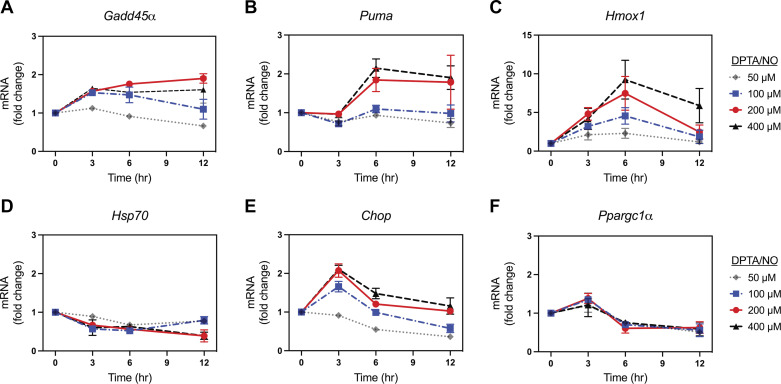
Concentration- and time-dependent effect of (*Z*)-1-[*N*-(3-aminopropyl)-*N*-(3-ammoniopropyl)amino]diazen-1-ium-1,2-diolate (DPTA/NO) on gene expression in mouse embryonic fibroblasts (MEF). MEF were treated with DPTA/NO for the indicated times and concentrations. The cells were harvested, and mRNA accumulation was determined by RT qPCR for *Gadd45α* (*A*), *Puma* (*B*), *Hmox1* (*C*), *Hsp70* (*D*), *Chop* (*E*), and *Ppargc1α* (*F*). Results are the means ± SE of three independent experiments.

We have recently identified several signaling cascades and metabolic pathways that are regulated by nitric oxide in a β-cell-selective manner (36, 39–41). To explore the cell type selectivity of nitric oxide on gene expression, the concentration- (50–400 μM) and time-dependent (0–12 h) effects of DPTA/NO on the accumulation of target gene mRNA were evaluated by qPCR in mouse embryonic fibroblasts (MEF). Much like INS 832/13 cells, 50 μM DPTA/NO has minimal effects on the mRNA accumulation of each of the six target genes examined (Fig. 6, *A*–*F*). At 200 and 400 µM DPTA/NO there is an ∼2-fold increase in *Gadd45α*, *Puma*, and *Chop* mRNA accumulation (Fig. 6, *A*, *B*, and *E*), whereas DPTA/NO does not stimulate *Hsp70* or *Ppargc1α* mRNA accumulation at any of the concentrations examined (Fig. 6, *D* and *F*). In fact, 200 and 400 μM DPTA/NO decrease the accumulation of *Hsp70* and *Ppargc1α* mRNA by ∼50% following a 12-h treatment (Fig. 6, *D* and *F*). The most responsive nitric oxide-dependent gene in MEF was *Hmox1*, where a 6-h incubation of 200 and 400 µM DPTA/NO stimulate a 7- and 10-fold increase, respectively (Fig. 6*C*). Unlike β-cells, there was no delay in expression of any of the genes examined following treatment with 200 and 400 µM DPTA/NO in MEF.

**Figure 7.**
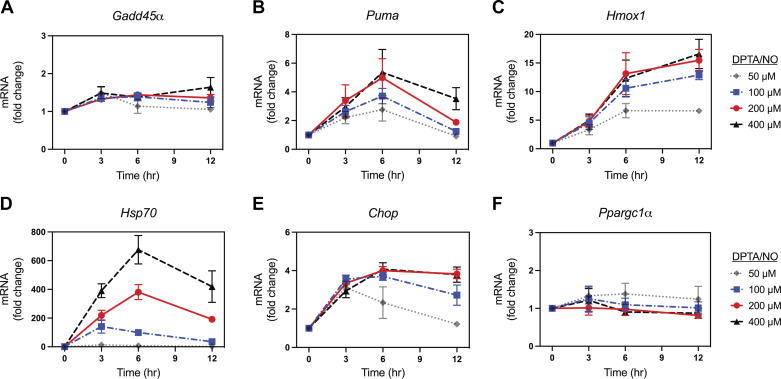
Concentration- and time-dependent effect of (*Z*)-1-[*N*-(3-aminopropyl)-*N*-(3-ammoniopropyl)amino]diazen-1-ium-1,2-diolate (DPTA/NO) on gene expression in αTC1 cells. αTC1 cells were treated with DPTA/NO for the indicated times and concentrations. The cells were harvested, and mRNA accumulation was determined by RT qPCR for *Gadd45α* (*A*), *Puma* (*B*), *Hmox1* (*C*), *Hsp70* (*D*), *Chop* (*E*), and *Ppargc1α* (*F*). Results are the means ± SE of three independent experiments.

The time- and concentration-dependent effects of nitric oxide on gene expression in glucagon expressing αTC1 cells (Fig. 7) were also examined. DPTA/NO fails to stimulate *Gadd45α* and *Ppargc1α* mRNA accumulation under any of the conditions examined (Fig. 7, *A* and *F*); however, this nitric oxide donor stimulates *Puma*, *Hmox1*, and *Hsp70* mRNA expression with a maximal increase of approximately 5-fold following a 6-h incubation for *Puma*, ∼15-fold following a 12-h incubation for *Hmox1*, and ∼700-fold following a 6-h incubation for *Hsp70* (Fig. 7, *B*–*D*). The actions of nitric oxide on *Hsp70* mRNA accumulation are concentration dependent and maximal at 400 µM (Fig. 7*D*), whereas *Puma* and *Hmox1* mRNA accumulation were concentration dependent and maximal at 200 µM DPTA/NO (Fig. 7, *B* and *C*). In response to 50–400 µM DPTA/NO, there is a 3- to 4-fold increase in *Chop* mRNA accumulation that remains elevated for 12 h (Fig. 7*E*). Unlike insulin containing cells (Figs. 3 and 4), we did not observe a delay in the expression of these genes in glucagon expressing αTC1 cells at higher nitric oxide concentrations.

**Figure 8.**
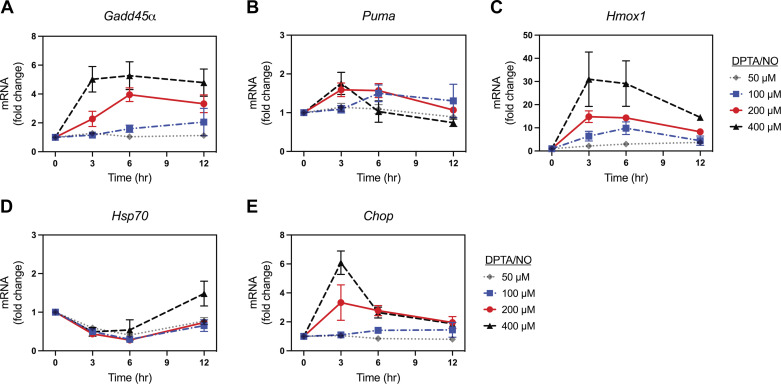
Concentration- and time-dependent effect of (*Z*)-1-[*N*-(3-aminopropyl)-*N*-(3-ammoniopropyl)amino]diazen-1-ium-1,2-diolate (DPTA/NO) on gene expression in RAW 264.7 cells. RAW 264.7 macrophages were treated with DPTA/NO for the indicated times and concentrations. The cells were harvested, and mRNA accumulation was determined by RT qPCR for *Gadd45α* (*A*), *Puma* (*B*), *Hmox1* (*C*), *Hsp70* (*D*), and *Chop* (*E*). Results are the means ± SE of three independent experiments.

RAW 264.7 macrophages represent the third non-β-cell in which the concentration- and time-dependent effects of nitric oxide on gene expression were examined (Fig. 8). In macrophages, nitric oxide does not stimulate *Hsp70* mRNA accumulation (Fig. 8*D*). Also, *Ppargc1α* mRNA accumulation in response to DPTA/NO is near or below the limit of detection of the assay (data not shown). In a concentration-dependent manner, DPTA/NO stimulates *Gadd45α* mRNA accumulation with maximal expression observed following 3–6 h exposure to 400 µM donor (Fig. 8*A*). Similarly, *Hmox1* and *Chop* mRNA accumulation increases in a concentration-dependent manner with maximal expression in response to a 3-h incubation with 400 µM DPTA/NO (Fig. 8, *C* and *E*). DPTA/NO had minimal effects on the accumulation of *Puma* mRNA (Fig. 8*B*). Like MEF and αTC1 cells, we did not observe any delays in the expression of target genes like those observed for β-cells treated with DPTA/NO at concentrations above 200 µM (Figs. 3 and 4). These findings suggest that the concentration-dependent actions of nitric oxide on gene expression are more tightly controlled in a narrow concentration range in β-cells as compared with the response of nonendocrine cells and α-cells.

### Role of Intermediary Metabolism in Regulating Nitric Oxide-Dependent Gene Expression in β-Cells

**Figure 9.**
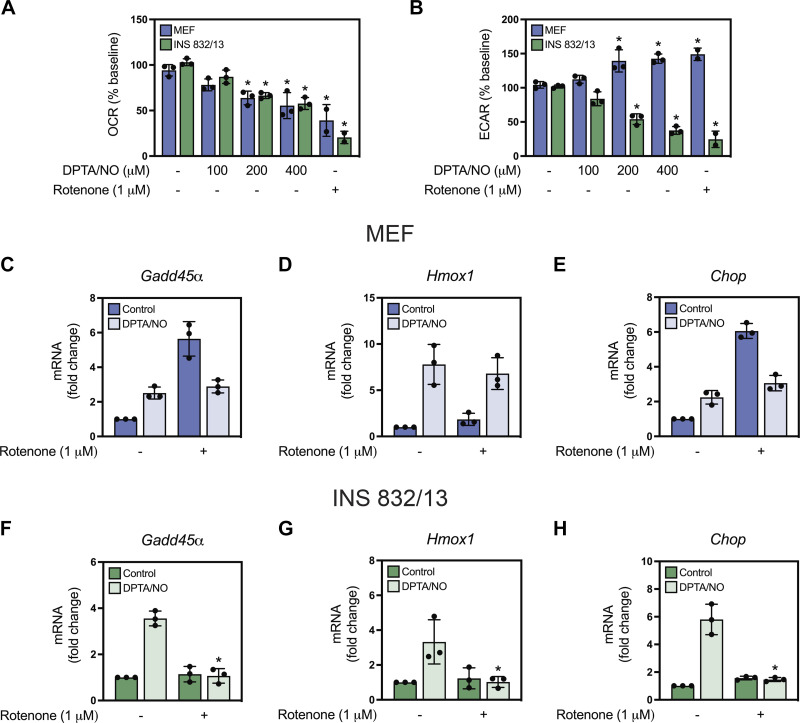
Effect of rotenone and (*Z*)-1-[*N*-(3-aminopropyl)-*N*-(3-ammoniopropyl)amino]diazen-1-ium-1,2-diolate (DPTA/NO) on mitochondrial respiration, glycolytic flux, and gene expression in mouse embryonic fibroblasts (MEF) and INS 832/13 cells. Intermediary oxidative metabolism was evaluated by extracellular flux analysis. Cells were treated at 15 min with the indicated concentrations of DPTA/NO or 1 µM rotenone, and the oxygen consumption rate (OCR, *A*) and extracellular acidification rate (ECAR, *B*) were determined after 120 min. The effects of rotenone on nitric oxide-stimulated gene expression in MEF and INS 832/13 cells were evaluated by RT qPCR. Cells were treated with stimulatory concentrations of DPTA/NO (200 µM, MEF, or 100 µM INS 832/13) for 3 h in the presence or absence of 1 µM rotenone, and mRNA accumulation of *Gadd45α* (*C* and *F*), *Hmox1* (*D* and *G*), and *Chop* (*E* and *H*) was determined by RT qPCR. Results are the means ± SE of two to three (*A* and *B*) or three (*C–H*) independent experiments. Statistically significant changes in OCR and ECAR relative to untreated control for each cell type (*A* and *B*) or mRNA accumulation for DPTA/NO plus rotenone relative to DPTA/NO alone (*C–H*) are as indicated (*P < 0.05).

We have recently identified several cellular pathways that are regulated by nitric oxide via its inhibitory actions on intermediary metabolism in a β-cell selective manner. We have shown that nitric oxide inhibits DDR signaling in insulinoma cells and isolated islets, and camptothecin-induced apoptosis of insulinoma cells by attenuating mitochondrial oxidation (26, 36), reducing ATP levels and inhibiting glucose uptake in β-cells (40). Inhibitors of mitochondrial oxidative metabolism function much like nitric oxide to attenuate each of these responses (41). To determine if the β-cell selective inhibition of gene expression at high concentrations of nitric oxide is associated with the inhibition of mitochondrial oxidative metabolism, we examined the effects of rotenone on gene expression in response to stimulatory concentrations of DPTA/NO. As expected, based on previous studies from our laboratory (40, 41), nitric oxide attenuates oxygen consumption (OCR) in a concentration-related manner in both INS 832/13 cells and MEF (Fig. 9*A*). MEF overcome this inhibition of mitochondrial oxidative metabolism by shifting to glycolysis for energy needs, as evidenced by increasing the extracellular acidification rate (ECAR; Fig. 9*B*). β-Cells lack this metabolic flexibility and fail to increase ECAR in response to inhibitors of mitochondrial oxidative metabolism (Fig. 9*B*). In fact, INS 832/13 cell ECAR decreases in response to rotenone (1 µM) and DPTA/NO at concentrations that limit OCR (200 and 400 µM), and we have previously attributed this action to decreases in ATP to levels that fall below the Km for ATP of glucokinase and result in an inhibition of glucose uptake (40, 41).

Consistent with the inhibition of mitochondrial oxidative metabolism as a mechanism by which 200 µM and 400 µM DPTA/NO fail to stimulate gene expression in β-cells after a 3-h incubation, rotenone (1 µM) attenuates DPTA/NO (100 µM) induced *Gadd45α*, *Hmox1*, and *Chop* expression by INS832/13 cells (Fig. 9, *F*–*H*). Rotenone does not attenuate DPTA/NO-stimulated expression of any of these genes in MEF (Fig. 9, *C*–*E*). Alone, rotenone increases *Gadd45α* and *Chop* mRNA accumulation independent of the actions of nitric oxide. These findings, which are consistent with the β-cell selective inhibitory actions of nitric oxide on DDR signaling and caspase 3 activation, suggest that high concentrations of nitric oxide (in excess of 1 µM measured or 2 µM calculated, Fig. 3, *G* and *H*) limit nitric oxide-dependent gene expression in a β-cell selective manner by a mechanism that is associated with the inhibition of mitochondrial oxidative metabolism.

## DISCUSSION

In 1985, Nerup and coworkers (8) first showed that IL-1 inhibits insulin secretion in a time- and concentration-dependent manner and that prolonged incubations of 4–7 days results in islet degeneration. Green and coworkers (17) first showed that these inhibitory actions of IL-1 are mediated by nitric oxide. Inhibitors of iNOS prevent the damaging actions of cytokines on insulin secretion (11, 18), and islets isolated from iNOS-deficient mice are resistant to cytokine-induced islet-cell death (56). Glucose-stimulated insulin secretion requires an increase in the ATP/ADP ratio that occurs in response to the oxidation of glucose to CO_2_ (57, 58). Nitric oxide inhibits the Krebs cycle enzyme aconitase and complex 4 of the electron transport chain, events that result in a decrease in the ATP/ADP ratio causing an inhibition in insulin secretion (13–16). Nitric oxide also induces DNA damage (59) and together with the inhibition of mitochondrial oxidative metabolism, nitric oxide is believed to be responsible for the loss of β-cell function and viability in response to IL-1 (60).

Based on this work, it is generally believed that cytokines such as IL-1 initiate or contribute to the loss of β-cell mass during the development of autoimmune diabetes (27); however, there are several lines of evidence that have led us to challenge this hypothesis. Islets have the capacity to recover from the damaging actions of IL-1 treatment if the cytokine is removed by washing and the islets are cultured in the absence of IL-1 for 4 days (30). Importantly, the time to complete recovery of insulin secretion following a 24-h IL-1 treatment can be reduced from 4 days to 8 h by inhibiting NOS, and this recovery occurs in the presence of IL-1 (31). In addition, β-cells (rat and human) maintain a FoxO1-dependent DNA repair pathway that is activated by nitric oxide and that requires GADD45α expression (33–35). Nitric oxide is also a strong activator of both the UPR and heat shock response (24, 61). When these responses are activated in β-cells, they become refractory to cytokine signaling (25, 62). These findings support a potential mechanism by which nitric oxide can temporally limit the inhibitory and damaging actions of cytokines on β-cells and allow for functional recovery.

The goal of the current study was to determine the concentration-dependent actions of nitric oxide on the expression of genes encoding proteins that participate in the physiological response of β-cells to this oxidant. We focused on six genes, the FoxO1-dependent genes *Gadd45α* and *Puma* (35), the NRF2-dependent gene *Hmox1*, the stress response genes *Hsp70* (heat shock) and *Chop* (unfolded protein response), and the master regulator of mitochondrial biogenesis *Ppargc1α* (43). The time- and concentration-dependent effects of nitric oxide supplied using the short half-life donor DEA/NO (∼3 min) and the longer half-life donor DPTA/NO (3 h) on the expression of each gene in INS 832/13 insulinoma, rat islets, αTC1 cells, MEF, and RAW 264.7 macrophages were examined. DEA/NO rapidly produces a bolus of nitric oxide (calculated to be ∼15 µM at 100 µM donor, and 45 µM when treated with 800 µM donor) that following 45 min of incubation results in steady-state levels of nitric oxide <1 µM. DPTA/NO releases nitric oxide at more physiological concentrations that were calculated to be ∼1 µM following a 3-h incubation with 50 µM DPTA/NO and ∼3.5 µM when supplied using 400 µM DPTA/NO. The calculated levels were higher than the levels directly measured using the NO analyzer, where we found that 50 µM DPTA/NO produces <0.5 µM nitric oxide following a 3-h incubation and >1 µM using 400 µM DPTA/NO.

**Figure 10.**
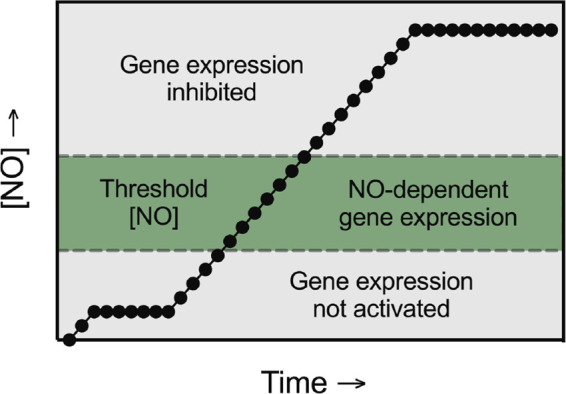
Concentration-dependent actions of nitric oxide on gene expression in β-cells. A schematic diagram illustrating the concentration- and time-dependent effects of nitric oxide on gene expression in β-cells.

Overall, we find that nitric oxide stimulates the expression of *Gadd45α, Puma*, *Hmox1*, and *Ppargc1α* in a narrow steady-state concentration range (∼0.5–1 µM measured, 1.5–2.5 µM calculated) in insulin-producing INS 832/13 cells and rat islets. Using donor concentrations that generate nitric oxide below the steady-state threshold, nitric oxide fails to stimulate gene expression. When nitric oxide is produced at steady-state levels above this threshold, the expression of each of these genes is temporally delayed until nitric oxide levels fall into this narrow concentration range. These unique findings are summarized in schematic fashion in Fig. 10. Furthermore, under conditions in which nitric oxide is produced at levels above the threshold, it is possible to scavenge this free radical using CPTIO and reduce the concentration to levels that fall within the threshold that facilitates gene expression (Fig. 5). This threshold action of nitric oxide appears to be selective for β-cells (Figs. 6, 7, and 8). DPTA/NO stimulates the concentration-dependent expression of *Gadd45α* in MEF and RAW 264.7 (Figs. 6*A* and 8*A*), *Puma* in MEF and αTC1 cells (Figs. 6*B* and 7*B*), and *Hmox1* in all three cell types (Figs. 6*C*, 7*C*, and 8*C*). *Ppargc1α* mRNA was not stimulated by nitric oxide in MEF or αTC1 cells (Figs. 6*F* and 7*F*) and was below the detection limits in RAW 264.7 (negative data not shown).

The expression of stress response genes (*Hsp70* and *Chop*) in β-cells in response to nitric oxide was found to behave slightly different (Figs. 3 and 4). DPTA/NO stimulates the concentration-dependent increase in *Hsp70* mRNA accumulation that is maximal between 9 and 12 h of treatment with 200 µM and 400 µM donor, respectively (Figs. 3*D* and 4*D*). Nitric oxide, a known UPR activator in β-cells, stimulates maximal *Chop* mRNA expression following a 6-h incubation with 100 and 200 µM DPTA/NO in INS 832/13 cells and rat islet cells, respectively (Figs. 3*E* and 4*E*). Increasing the concentration of DPTA/NO to 200 and 400 µM (INS 832/13) or 400 µM (rat islets) delayed the accumulation of *Chop* mRNA and decreased maximal stimulation (Figs. 3*E* and 4*E*). These findings indicate that nitric oxide activates UPR gene expression in β-cells when produced at steady-state calculated concentrations of 1.5–2.5 µM or measured concentrations of ∼0.5 µM, and that the dampening of *Chop* expression at higher nitric oxide concentrations suggests that the β-cell response to misfolded proteins may shift from the UPR toward a heat shock response with increasing concentrations of nitric oxide. In support of this possibility, *Hsp70* mRNA increases ∼550-fold in INS 832/13 cells and ∼65-fold in dispersed rat islets after treatment with 400 μM DPTA/NO (Figs. 3*D* and 4*D*). We also observed cell-type specificity in the activation of stress responses. Although nitric oxide stimulated *Chop* mRNA accumulation in all cell types (Figs. 3, 4, 6, 7, and 8), the induction of the heat shock response appears to be an endocrine cell-selective event. Nitric oxide does not stimulate *Hsp70* mRNA accumulation in MEF (fibroblast) or RAW 264.7 macrophages (Figs. 6 and 8); however, insulin and glucagon containing cells respond to nitric oxide with robust expression of *Hsp70* (Figs. 3, 4, and 7). These findings are consistent with single-cell RNA-seq analysis of cytokine and nitric oxide stimulated changes in gene expression in dispersed mouse islet cells, where iNOS-derived nitric oxide increased *Hsp70* mRNA in endocrine cells but failed to do so in nonendocrine cell populations (52).

Nitric oxide binds to heme-containing proteins (63) and has been shown to stimulate expression of NRF2-dependent genes, such as *Hmox1* (64, 65). Once expressed, heme oxygenase (HO)-1 enzymatically degrades heme to biliverdin, ferrous iron, and carbon monoxide (66). In all cell types examined, including β-cells, nitric oxide stimulates *Hmox1* mRNA accumulation (Figs. 3, 4, 6, 7, and 8). This ubiquitous expression of *Hmox1* is consistent with the stabilization of NRF2 in response to numerous oxidants (67), which allows for NRF2 nuclear translocation, binding to antioxidant response elements (AREs), and transcription of antioxidant genes (68).

Nitric oxide inhibits mitochondrial function through disruption of iron-sulfur clusters of aconitase and complex IV of the electron transport chain by competing with oxygen binding (13–16). Despite its inhibitory actions on mitochondrial function, nitric oxide stimulates mitochondrial biogenesis (69), in part, by the increased expression of peroxisome proliferator-activated receptor gamma coactivator 1α, an important transcriptional coactivator that regulates genes involved in mitochondrial biogenesis (43, 53). We report that *Ppargc1α* gene expression is delayed in β-cells when nitric oxide is generated at calculated concentrations above 3 μM or measured concentrations of 1 µM (Figs. 3*F* and 4*F*). The delay of *Ppargc1α* expression at higher DPTA/NO concentrations (200 and 400 µM) correlates with an inhibition of mitochondrial oxidative metabolism and loss of ATP (39–41). The increased expression of *Ppargc1α* at concentrations of DPTA/NO that do not inhibit mitochondrial oxidative metabolism may function to protect mitochondria from higher concentrations of nitric oxide that limit oxidative metabolism, and/or may facilitate the recovery of metabolic function when the levels of nitric oxide fall below the concentrations that limit mitochondrial oxidative metabolism (31, 70).

### Perspectives and Significance

It is well established that nitric oxide causes DNA damage in β-cells (33, 59), and we have shown that β-cells utilize the BER pathway gene, *Gadd45α*, to repair DNA damage in response to nitric oxide (34). Nitric oxide stimulates *Gadd45α*, in a FoxO1-dependent manner (35). FoxO1 is also required for nitric oxide-stimulated *Puma* expression in β-cells (35). Puma binds antiapoptotic Bcl-2 family members, allowing proapoptotic proteins such as Bax/Bak to initiate cytochrome C release from the mitochondria to activate caspases (71). Both *Gadd45α* and *Puma* mRNA accumulation were observed after a 6-h treatment of INS 832/13 cells with 100 μM DPTA/NO, and expression is delayed at higher nitric oxide concentrations (Fig. 3, *A* and *B*). We view these findings to suggest that β-cells are prepared for two responses. One is base excision repair of damaged DNA (*Gadd45α*) and subsequent recovery, or if the DNA damage is too extensive for repair, apoptosis (*Puma*). Although somewhat controversial in the literature, it has been our experience (33, 72) and that of others (56, 73, 74), that it is challenging to induce apoptosis in primary β-cells in response to cytokines or nitric oxide. In fact, nitric oxide inhibits apoptosis in insulinoma cells in response to apoptosis activators (36) or chemical endoplasmic reticulum (ER) stress inducers, such as tunicamycin (24).

Recently, we have shown that nitric oxide inhibits DDR signaling (ATM, ataxia telangiectasia mutated and ATR, ataxia telangiectasia and Rad3-related) by a mechanism that is associated with the inhibition of mitochondrial oxidative metabolism and decreases in cellular levels of ATP (39, 41). Nitric oxide reduces the concentrations of ATP in β-cells to levels that are 2-fold below the *K*_mATP_ of glucokinase resulting in an inhibition in glucose uptake (40). The inhibitory actions of nitric oxide on glucose uptake are selective for β-cells (40, 41), which we believe functions as a physiologically relevant protective mechanism. In support of this hypothesis, we have shown that nitric oxide inhibits picornavirus replication in β-cells by attenuating mitochondrial oxidative metabolism (37, 75). Importantly, the inhibitory actions of nitric oxide on oxidative metabolism are completely reversible (31), and we hypothesize that the inhibition of mitochondrial oxidative metabolism by nitric oxide, while appearing to be a damaging action due to the inhibition of insulin secretion, may function as a protective host-defense mechanism limiting virus replication and attenuating β-cell death by apoptosis. At concentrations that do not inhibit mitochondrial oxidative metabolism (∼1.5–2.5 µM calculated or 0.5–1 µM measured) nitric oxide stimulates the expression of genes that participate in the recovery of metabolic and secretory function (*Gadd45α*, *Ppargc1α*), the defense against oxidant damage (*Hmox1*), and functions as an off switch limiting cytokine signaling by activating stress responses (evidenced by *Hsp70* and *Chop* expression). We and others have shown that heat shock, and islets expressing the inducible heat shock protein HSP70 are resistant to cytokine signaling (25, 52, 62, 72, 76). These protective actions are stimulated by sustained production of nitric oxide at concentrations calculated to be ∼1.5–2.5 μM or measured levels of 0.5–1 µM, or iNOS-derived levels of nitric oxide (Fig. 10). When the concentration increases above these levels, nitric oxide inhibits mitochondrial oxidative metabolism and limits protective gene expression until the levels of this free radical dissipate into the stimulatory concentration range (Fig. 10). The concentration-dependence of nitric oxide-stimulated gene expression is a phenomenon that appears to be selective to β-cells and is associated with several protective responses that include the induction of DNA repair, the activation of mitochondrial biogenesis pathways, attenuation of virus replication, and the inhibition of β-cell apoptosis.

## DATA AVAILABILITY

Data will be made available upon reasonable request.

## GRANTS

This work was supported by the National Institute of Diabetes and Digestive and Kidney Diseases Grant R01DK052194 (to J.A.C.), by the National Institute of Allergy and Infectious Diseases Grant R01AI044458 (to J.A.C.), the Juvenile Diabetes Research Foundation Grant Key: 3-SRA-2023-1281-S-B, and gifts from the Scott Tilton Foundation and Forest County Potawatomi Foundation (to J.A.C.).

## DISCLOSURES

No conflicts of interest, financial or otherwise, are declared by the authors.

## AUTHOR CONTRIBUTIONS

A.N., C.T.Y., and J.A.C. conceived and designed research; A.N., C.T.Y., and N.H. performed experiments; A.N., N.H., and J.A.C. analyzed data; A.N., C.T.Y., and J.A.C. interpreted results of experiments; A.N. prepared figures; A.N. and J.A.C. drafted manuscript; A.N., C.T.Y., and J.A.C. edited and revised manuscript; A.N., C.T.Y., N.H., and J.A.C. approved final version of manuscript.
